# Evaluating AI in leukocyte classification: performance of the AI system against 15 morphology experts

**DOI:** 10.1038/s41746-026-02601-w

**Published:** 2026-04-11

**Authors:** Wei Xu, Haoqin Jiang, Jingxian Zhang, Kun Chen, Yang Fei, Ping Guo, Yongjian He, Yi Ye, Linlin Qu, Mingkang Yang, Lizhi Yan, Di Wang, Huan Qi, Shihong Zhang, Chi Zhang, Jianbiao Wang, Ming Guan

**Affiliations:** 1 Department of Laboratory Medicine, The First Bethune Hospital of Jilin University, Jilin, China; 2 Department of Laboratory Medicine, Huashan Hospital Fudan University, Shanghai, China; 3 Clinical Department (IVD), Shenzhen Mindray Bio-Medical Electronics Co. Ltd., Shenzhen, China; 4 Department of Laboratory Medicine, Tongji Hospital, Tongji Medical College of Hust, Wuhan, China; 5 Department of Laboratory Medicine, Ruijin Hospital, Shanghai Jiaotong University School of Medicine, Shanghai, China; 6 Department of Laboratory Medicine, Nanfang Hospital, Guangzhou, China; 7 Department of Laboratory Medicine, The First Affiliated Hospital Sun Yat-sen University, Guangzhou, China

**Keywords:** Biomarkers, Computational biology and bioinformatics, Diseases, Immunology, Medical research

## Abstract

Artificial intelligence (AI)-based digital morphology analyzers are vital for clinical hematology but face critical challenges in identifying immature granulocytes and atypical cells. This study validated the MC-100i AI system against 15 morphologists, with an additional 3 senior experts establishing a gold standard, to assess performance gaps and clinical utility. A total of 104 blood smears containing 19,174 cells (9 abnormal types, including 3154 leukocytes and nucleated erythrocytes) were analyzed. The AI achieved an overall accuracy of 95.97% (ranked second) and 91.38% accuracy for the abnormal subsets (ranked fifth). Critical biases were identified: AI classified ambiguous cells (e.g., promyelocytes) as earlier developmental stages, while humans favored later stages. AI relied on isolated features for atypical cells, unlike experts who integrated smear context. These findings confirm the AI’s robust clinical potential for routine leukocyte classification, and targeted optimizations will further enhance AI-driven peripheral blood cell identification for effective integration into clinical diagnostic workflows.

## Introduction

Peripheral blood leukocyte analysis is essential for disease diagnosis and management^1,2^. Given that manual microscopy remains the gold standard despite its limitations, such as labor intensity, time consumption, and lack of standardized image storage^3,4^ have driven the development of automated digital morphology analyzers since the 1960s^5–7^

Advances in artificial intelligence (AI) technology have resulted in fast, accurate, standardized, and cost-effective expert opinions for telehaematology through remote, real-time conferencing^8^. Many clinical laboratories have comprehensively evaluated and introduced digital cell morphology analysers for peripheral blood cells in routine clinical practice, and their excellent performance in identifying peripheral blood leukocytes and alerting for abnormal samples has been widely reported. However, the identification accuracy for some highly heterogeneous cells (such as abnormal lymphocytes and immature granulocytes) remains lacking^7,9–12^. We speculate that this may be due to the diversity and substantial morphological variability among some cells and the susceptibility to morphological differences^13^. Studies revealed large differences among morphology experts in the identification of leukocytes under manual microscopy^14,15^, These differences pose challenges to evaluating the identification accuracy and performance of digital cell morphology analysers.

The excellent pre-classification performance of digital cell morphology analysers has led to their widespread use in clinical practice. Nevertheless, this study aimed to investigate the causes of misidentification of partial cell. If it is understood that the identification of abnormal cells by different morphology experts is affected by subjective factors, then what are the identification preferences of digital cell morphology analysers? In this study, the cell identification performance of the MC-100i was compared with that of 15 morphology experts to demonstrate the cell identification capability of the analyser’s AI system with respect to different morphology experts and to elucidate the preferential recognition of the MC-100i in the classification of peripheral blood cells. Further, an in-depth discussion of the reasons for the low identification rates of the AI system for certain cells is provided, identifying factors influencing accuracy to guide future improvements.

## Results

### Performance of the AI system vs. 15 morphology experts in identifying 14 types of leukocytes and nucleated erythrocytes

**Fig. 1:**
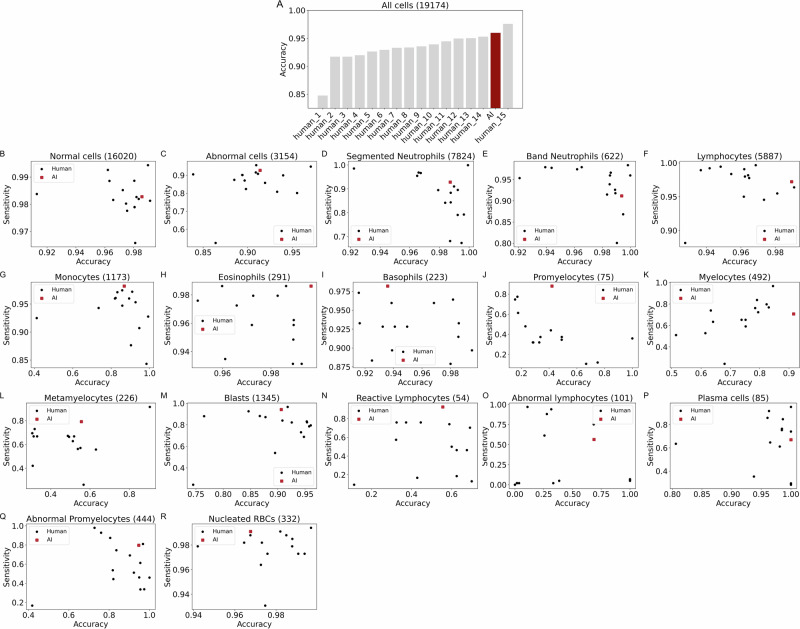
Classification performance of 15 morphologists and the AI system for 14 types of leukocytes and nucleated erythrocytes. (**A**) Overall accuracy across 19,174 cells (gray: human experts, red: AI).(**B**–**R**) Bivariate sensitivity-accuracy plots for normal/abnormal hematopoietic cell subsets (cell counts annotated): (**B**) Normal cells, (**C**) Abnormal cells, (**D**) Segmented neutrophils, (**E**) Band neutrophils, (**F**) Lymphocytes, (**G**) Monocytes, (**H**) Eosinophils, (**I**) Basophils, (**J**) Promyelocytes, (**K**) Myelocytes, (**L**) Metamyelocytes, (**M**) Blasts, (**N**) Reactive lymphocytes, (**O**) Abnormal lymphocytes, (**P**) Plasma cells, (**Q**) Abnormal promyelocytes, (**R**) Nucleated red blood cells. Black dots: individual morphologists; red dot: MC-100i AI. This figure quantifies and compares the classification accuracy of 15 morphologists and the MC-100i AI system across 15 distinct cell types, which are grouped into normal cells (band neutrophils, segmented neutrophils, eosinophils, basophils, lymphocytes, monocytes) and abnormal cells (blasts, promyelocytes, myelocytes, metamyelocytes, reactive lymphocytes, abnormal lymphocytes, abnormal promyelocytes, plasma cells and nucleated erythrocytes).

A total of 19,174 cells from 104 samples were included in this study. The classification results showed that one morphological expert accurately identified 18,710 cells with an accuracy of 97.58%, ranking first; the AI system accurately identified 18,402 cells with an accuracy of 95.97%, ranking second (Fig. 1A). To further quantify and verify the difference in classification performance between AI and each morphologist, based on the accuracy ranking, this study conducted a rate difference confidence interval analysis, comparing the cell recognition accuracy of AI with that of 15 morphologists one by one. The criterion for this analysis was: if the lower limit of the 95% confidence interval of the rate difference (AI accuracy – morphologist accuracy) was higher than the superiority margin of 0, the difference in classification results between AI and the morphologist was considered statistically significant. The paired analysis results showed that the lower limits of the 95% confidence intervals of the rate differences between AI and 14 morphologists were all greater than 0, indicating that the classification results of AI were superior to those 14 morphologists; among them, the lower limit of the 95% confidence interval of the rate difference between AI and one morphologist was greater than 10%, indicating that AI showed significant superiority over this morphologist. Detailed classification results are shown in Table 1.

**Table 1:** Analysis Table of Confidence Intervals for Rate Differences in Classification Performance: AI vs. Morphologists

Personnel type	AI accuracy	Morphologist accuracy	Rate Difference (AI-morphologist)	95% CI lower limit	95% CI upper limit
Human_1	95.97%	84.76%	11.21%	10.74%	11.69%
Human_4	95.97%	91.98%	3.99%	3.71%	4.29%
Human_3	95.97%	91.71%	4.27%	3.94%	4.60%
Human_5	95.97%	92.63%	3.35%	3.07%	3.64%
Human_6	95.97%	92.93%	3.04%	2.79%	3.30%
Human_2	95.97%	91.70%	4.28%	3.96%	4.61%
Human_12	95.97%	94.96%	1.01%	0.76%	1.27%
Human_13	95.97%	95.02%	0.95%	0.68%	1.24%
Human_15	95.97%	97.58%	−1.61%	−1.95%	-1.27%
Human_14	95.97%	95.29%	0.69%	0.45%	0.93%
Human_9	95.97%	93.55%	2.43%	2.19%	2.67%
Human_10	95.97%	93.92%	2.05%	1.88%	2.24%
Human_8	95.97%	93.35%	2.63%	2.26%	3.00%
Human_7	95.97%	93.30%	2.68%	2.32%	3.04%
Human_11	95.97%	94.45%	1.52%	1.23%	1.82%

Subgroup analysis of cell types showed that the AI system achieved an identification accuracy of 98.57% for 6 types of normal leukocytes (Fig. 1B); for 9 types of clinically relevant abnormal cells (metamyelocytes, myelocytes, promyelocytes, blast cells, reactive lymphocytes, plasma cells, abnormal lymphocytes, abnormal promyelocytes, and nucleated red blood cells), the AI system achieved an identification accuracy of 91.38%, which was superior to 10 morphologists, with a detection rate of 92.74% (Fig. 1C).

The results of F1 score analysis (see Supplementary Table S1) indicated that there were differences in classification performance between the AI system and morphologists on different types of cells. For normal leukocytes (such as segmented neutrophils, lymphocytes, etc.), the F1 scores of both AI and morphologists were mostly at a high level. Among them, the F1 scores of AI on lymphocytes and eosinophils were 0.981 and 0.991, respectively, slightly higher than those of most morphologists; among abnormal cells, AI showed excellent F1 scores on nucleated red blood cells and blast cells (0.924 and 0.979, respectively), which were superior to most morphologists; while in immature granulocytes, the F1 scores of both AI and most morphologists were not high, ranging from 0.302 to 0.798. Subsequent subsections will conduct a detailed analysis of the identification results based on the morphological characteristics of various cells identified by the AI system.

### Six types of normal leukocytes: AI system vs. 15 morphology experts

The 15 morphology experts and the AI system all achieved an accuracy of over 90% in identifying 16,020 normal cells; achieving 98.57% overall accuracy, and outperforming 13 morphology experts (Fig. 1B). The AI system achieved an identification accuracy of over 98% for band neutrophils, segmented neutrophils, lymphocytes, and eosinophils (Figs. 1D–F, 3H); It showed particular strength in recognizing eosinophils (99.65% accuracy), surpassing all human experts (Fig. 1H). However, in identifying monocytes, the AI system achieved an identification accuracy of 86.75%, similar to that of 9 morphology experts (less than 90%) (Fig. 1G), likely due to their morphological variability. Overall, the identification accuracy and sensitivity of the AI system in identifying these six types of cells exceeded those of some morphology experts.

**Fig. 2:**
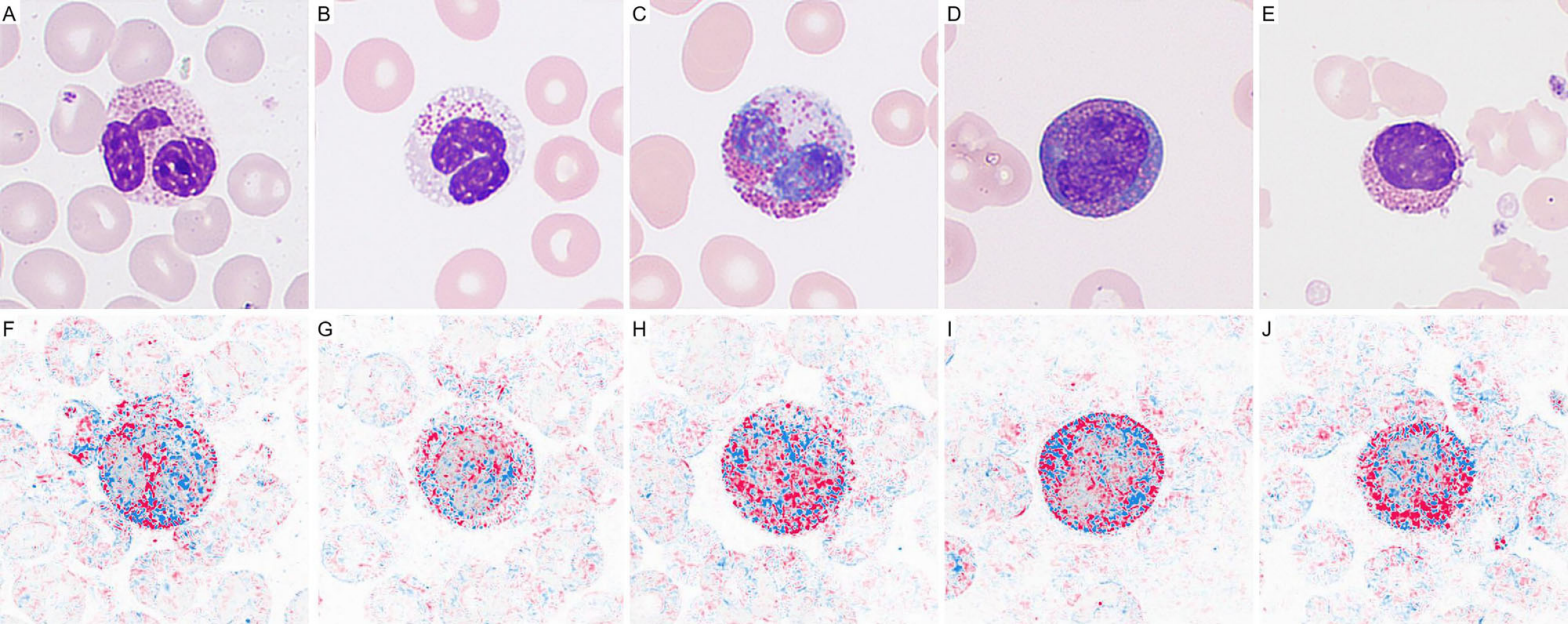
Eosinophils misjudged by morphologists and Artificial Intelligence (AI) feature recognition. This figure displays (**A**–**E**) morphological images of misclassified eosinophils with distinct phenotypes (sparse or abnormally stained granules in (**A**–**C**), immature forms in (**D**, **E**) and (**F**–**J**) SHapley Additive exPlanations (SHAP) analyses that visualize how the AI system captures eosinophilic granule features during pre-classification, thereby revealing the phenotypic and algorithmic underpinnings of misclassification by both human morphologists and the AI system.

The superiority of the AI system over some morphological experts in identifying normal leukocytes (band neutrophils, segmented neutrophils, lymphocytes, monocytes, basophils, and eosinophils), likely due to these cells’ distinct morphological features and larger training datasets. For example, eosinophils have a cytoplasm filled with large, uniform, and orange‒red eosinophilic granules, distinct from the cytoplasmic constitution of other normal cells. The AI system correctly identified all cells shown in Fig. 2 based on their prominent and consistent features. Specifically, SHapley Additive exPlanations (SHAP) analysis revealed that the AI system effectively captured eosinophilic granules in different cells during pre-classification, as shown in Fig. 2F–J. Figure 2A–C have few or abnormally colored eosinophilic granules; in contrast, some morphologists misclassified these three cells as segmented neutrophils, basophils, or monocytes, possibly due to human errors when reviewing large sample volumes or misclassification when the features are not obvious. Additionally, Fig. 2D, E were identified as immature eosinophils. Although we standardized the classification before the study and included immature eosinophils under eosinophils, some morphologists still classified them as myelocytes.

### Immature granulocytes in blood cell development: AI system vs. 15 morphology experts

**Fig. 3:**
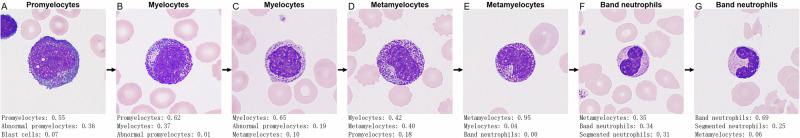
The top three predicted scores of the AI system for cells in developmental stages. This figure presents morphological images of granulocytes across sequential developmental stages, spanning from promyelocytes (**A**) to band neutrophils (**G**). Panels A–G collectively cover the full maturation trajectory: **A** promyelocytes, **B**–**F** representative intermediate stages including myelocytes, metamyelocytes, and segmented neutrophils, and **G** band neutrophils. Each image is paired with the AI system’s top three predicted cell types and their corresponding confidence scores, illustrating the model’s classification patterns and potential systematic biases in identifying maturing granulocyte populations.

The AI system demonstrated inconsistent performance in identifying immature granulocytes. It had low identification accuracies in identifying 75 promyelocytes and 226 metamyelocytes but sensitivities of 88% and 79.20%, respectively (Figs. 1J, 3L). The system achieved an identification accuracy of 91.58% (ranking first) and a sensitivity of 70.73% for 492 neutrophilic myelocytes, and an identification accuracy of 99.58% but a sensitivity of only 87.23% (Fig. 1K).

These inconsistencies stem from inherent challenges in classifying developing granulocytes, where three key factors contribute to diagnostic ambiguity: (1) cytoplasmic granules frequently obscure nuclear morphological changes, (2) the transitional nature of granules makes it difficult to distinguish between non-specific and specific granule types, and (3) the continuous spectrum of cellular maturation lacks clear-cut boundaries between developmental stages. These objective morphological complexities, combined with the subjective interpretation thresholds of individual morphologists, create significant variability in cell stage classification.

The analysis revealed significant classification discrepancies for developing granulocytes. Notably, none of the 75 promyelocytes received unanimous identification from all 15 experts and the AI system. As shown in Fig. 3, while the gold standard and 9 morphologists classified cell B as a myelocyte, the AI system and 6 morphologists identified it as a promyelocyte. This cell exhibited an oval nucleus obscured by granules, making it impossible to clearly discern nuclear chromatin details or determine whether the granules were the neutral granules of myelocytes or the more abundant A granules of promyelocytes. Consequently, morphologists struggled to distinguish between late-stage promyelocytes and early-stage myelocytes. The AI system also showed uncertainty, predicting a 0.62 probability for promyelocyte and 0.37 for myelocyte. Similar disagreements occurred with metamyelocytes (Fig. 3D) and band neutrophils (Fig. 3F), showing a systematic pattern: experts tended to classify borderline cells into later developmental stages, while the AI conservatively assigned earlier stages to minimize missed detections. (as shown in Fig. 3).

### Atypical cells with similar morphologies: AI system vs. 15 morphology experts

**Fig. 4:**
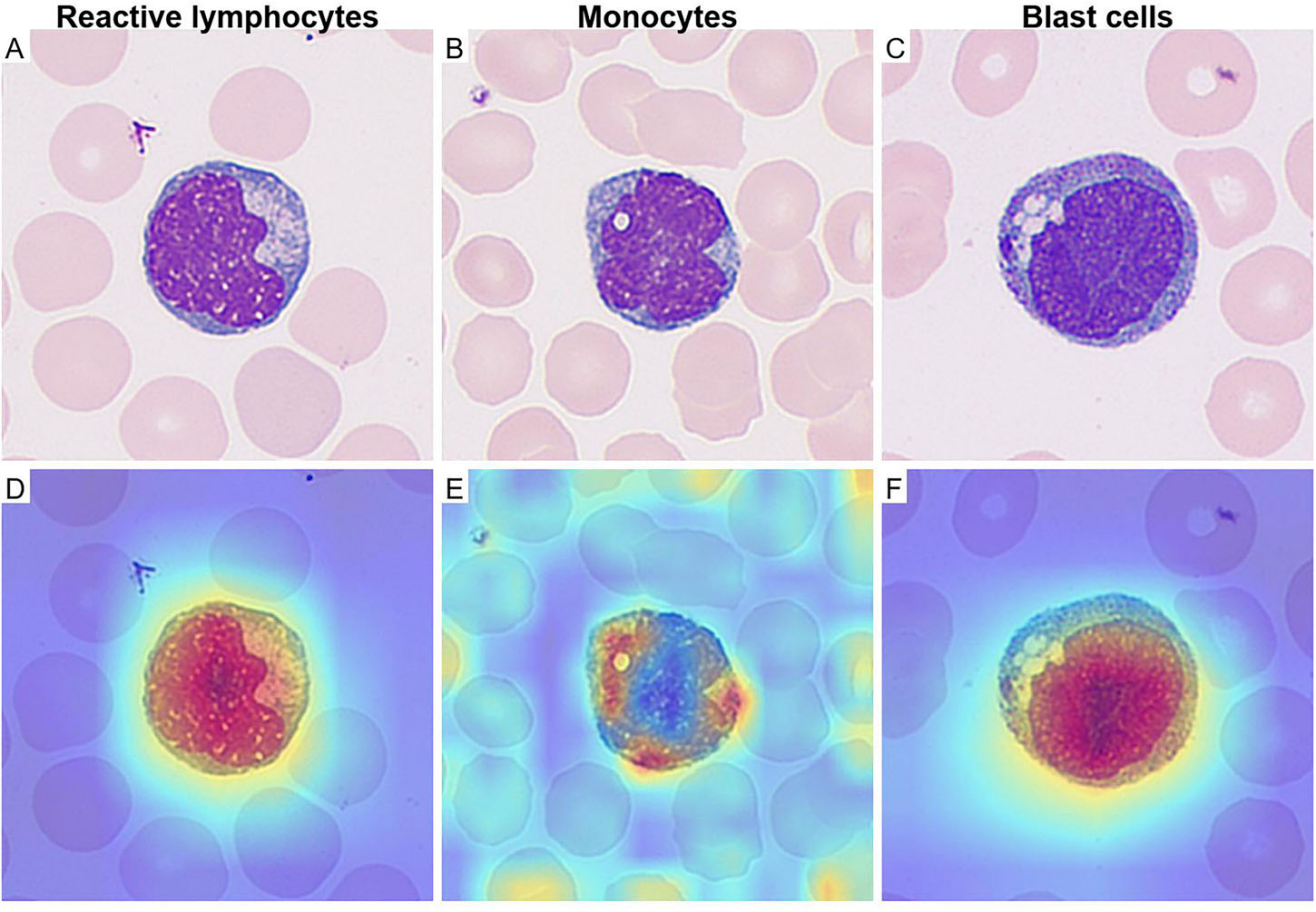
Cells with divergent morphologist judgments and AI classification interpretability via Grad-CAM analysis. This figure presents **A**–**C** morphological images of three cell types with divergent recognition among morphologists (Cell A: reactive lymphocyte; Cell B: monocyte; Cell C: blast cell) and **D**–**F** the corresponding Grad-CAM heatmaps for the AI model. In the heatmaps, red regions indicate high-importance areas used by the AI model for classification, while blue regions represent low-importance areas.

The AI system demonstrated high sensitivity but moderate accuracy in classifying morphologically similar atypical cells - achieving 98.21% sensitivity/86.75% accuracy for monocytes, 94.20%/90.63% for blast cells, and 92.59%/55.56% for reactive lymphocytes (Fig. 1G, M, N). This pattern reflects the system’s conservative approach to avoid missed detections, particularly evident in its tendency to classify borderline stimulated lymphocytes as reactive lymphocytes rather than normal lymphocytes per the gold standard. The significant morphological variability of reactive lymphocytes (especially types II and III) and their overlapping features with monocytes and blast cells contribute to these challenges. As shown in Fig. 4, even experts disagreed on classifications: a reactive lymphocyte with monocytoid features (Cell A) was misclassified as a monocyte by both AI and 10 experts, while a monocyte (Cell B) was misidentified as a reactive lymphocyte by AI and 5 experts, and a blast cell (Cell C) was incorrectly labeled as a monocyte by AI and 3 experts. Corresponding Gradient-weighted Class Activation Mapping (Grad-CAM) analyses are presented in Fig. 4D–F to visualize the key morphological regions focused on by the AI model. These discrepancies highlight how the AI’s single-cell analysis paradigm, while effectively capturing salient morphological features, lacks the contextual smear evaluation and multi-cell assessment that experts employ when interpreting ambiguous cases. The findings suggest that incorporating whole-smear analysis and clinical correlation could enhance future AI systems’ performance on these challenging cell types.

### Four other types of abnormal cells: AI system vs. 15 morphology experts

The AI system showed strong performance for certain abnormal cells, particularly for nucleated erythrocytes (96.76% accuracy, 99.10% detection rate; Fig. 1R) and abnormal promyelocytes (94.65% accuracy; Fig. 1Q), outperforming most morphologists. However, its 79.73% detection rate for treated abnormal promyelocytes reflected challenges in classifying these morphologically heterogeneous cells during treatment transitions, where the AI’s single-cell analysis differed from experts’ comprehensive sample evaluation.

For the more challenging abnormal lymphocytes and plasma cells, the system achieved 68.67% and 56.44% accuracy, respectively (Fig. 1O, P). The limited sample sizes (2 abnormal lymphocyte and 4 plasma cell cases) suggest these results should be interpreted cautiously, though the 100% sensitivity for plasma cells remains notable.

## Discussion

Peripheral blood smear analysis remains crucial for diagnosing and monitoring hematological disorders^2,16^, though traditional manual microscopy suffers from subjectivity and inefficiency^17,18^. Although current-generation digital analyzers (such as those from Mindray and Cellavision) have been clinically validated^10,19^, the field must progress toward deeper interrogation of AI interpretation patterns that transcend simple accuracy measurements.

Our MC-100i’s CNN-based system progressively extracts hierarchical image features^16^, enabling precise cell identification that overcomes several key limitations of human analysis. The system’s performance advantages were particularly evident in three critical areas: First, in eosinophil classification, where the AI consistently recognized characteristic features that some morphologists missed, correctly identifying cells that were misclassified as segmented neutrophils, monocytes, or basophils by human experts. Second, the system maintained this precision even for rare immature eosinophils that were frequently miscategorized as myelocytes despite standardized review criteria. This result highlights how regional classification variations among morphologists can compromise diagnostic consistency, and reducing such misclassifications is of great significance for ensuring diagnostic accuracy. Third, and most clinically significant, the system showed particular strength in identifying rare abnormal promyelocytes (surpassing 11 of 15 morphologists), which is crucial for timely Acute Promyelocytic Leukemia (APL) diagnosis, where treatment delays are known to increase mortality^20^. and misclassification of abnormal promyelocytes (e.g., misjudged as blast cells or typical promyelocytes) is one of the key factors leading to diagnostic delays. Therefore, the system’s accurate identification of these cells can directly avoid such misclassification risks.

The superior performance in abnormal promyelocyte detection deserves particular emphasis. While less-experienced morphologists often misclassify these cells as blasts or typical promyelocytes due to their rarity and morphological complexity, the AI’s data-driven approach enables accurate identification through large-scale pattern recognition from training datasets. This capability directly supports early All-Trans Retinoic Acid (ATRA) intervention, potentially reducing APL mortality through prompt treatment initiation^20^ Our comparative analysis revealed fundamental differences in classification approaches that help explain these performance advantages. The AI demonstrated a consistent tendency to classify borderline cells as earlier developmental stages, resulting in higher sensitivity for abnormal cell detection (albeit with more false positives), while human experts more frequently assigned later-stage classifications. This divergence was most pronounced in promyelocyte identification, where complete diagnostic consensus proved unattainable among the 15 participating morphologists and the AI system.

The above findings carry several important implications for clinical practice directly related to the prevention and control of misclassifications: First, given the inherent subjectivity in developmental staging, sample-level analysis may provide more reliable diagnostic information than individual cell classification when evaluating immature granulocytes. This helps reduce diagnostic biases caused by misjudgment of individual cell staging. Second, although the AI’s classification tendency favors abnormal categorizations, for time-sensitive conditions like APL, the benefits of early detection (i.e., reducing missed diagnoses caused by misclassification of abnormal cells) clearly outweigh the costs of over-identification, which provides a reference for clinically balancing the risk of misclassification and the timeliness of diagnosis and treatment ^21^.

However, our study also highlights important limitations that must be acknowledged. For example, relatively few plasma cell samples were included (only 57 cells from four cases), meaning that while the identification accuracy for these cells was 100%, this result may not be fully representative of the AI’s capabilities with this cell type. More broadly, the classification of atypical cells - particularly monocytes, blast cells, and reactive lymphocytes - continues to present significant challenges for both AI systems and human morphologists due to their inherent morphological complexity and variability. As recognized by the European LeukemiaNet classification scheme^15^ even expert morphologists require comprehensive contextual analysis of surrounding cells to accurately identify these cell types, as their appearance can vary substantially across different samples and disease states.

These findings carry several important implications for clinical practice. First, they suggest that sample-level analysis may provide more reliable diagnostic information than individual cell classification when evaluating immature granulocytes, given the inherent subjectivity in developmental staging. Second, the AI’s conservative classification strategy - while generating more potential false positives - offers particular clinical value for time-sensitive conditions like APL, where early detection outweighs the costs of over-identification.

Technical variables in sample preparation and imaging - including staining methods, resolution, magnification, and other pre-analytical factors^1^-represent another important consideration. While our study employed rigorously controlled preparation methods using the SC-120 system to minimize these variables, broader clinical implementation of AI-assisted morphology analysis will require comprehensive standardization efforts comparable to those established in radiology through Digital Imaging and Communications in Medicine (DICOM)^16^. This includes developing uniform protocols for image acquisition, data formatting, and annotation procedures to ensure consistency across institutions. The parallel development of large-scale, expertly annotated databases incorporating diverse disease states and treatment phases represents another essential requirement for advancing the field, though such initiatives inevitably face substantial practical challenges in terms of resource requirements and case collection. Notably, the AI’s data-driven approach demonstrates unique potential to overcome human subjectivity in morphological interpretation while potentially identifying novel, clinically-relevant cellular features through advanced dimensional analysis ^22,23^.

Ultimately, our results demonstrate that AI systems can serve as valuable clinical tools when these standardization challenges are adequately addressed, complementing rather than replacing expert morphological assessment. The path forward requires coordinated efforts to establish robust technical standards, implement rigorous quality control measures, and develop integrative platforms that combine cellular morphology with other relevant clinical data. Such advancements will enable more reliable and clinically actionable AI-assisted hematological diagnosis while preserving the essential role of expert interpretation in complex cases. By combining the strengths of AI-based pattern recognition with human clinical expertise, we can work toward more accurate, efficient, and standardized approaches to peripheral blood morphology analysis that benefit both clinicians and patients.

## Methods

### Cell Image Bank Construction

The study subjects included 104 patients who were admitted to Ruijin Hospital between March 20, 2022, and April 1, 2022. Supplementary Table S2 shows the distribution of the 19,174 total cells identified from the samples of the 104 patients, demonstrating that these cells adequately covered the 15 peripheral blood cell types of interest. Of the patient’s blood samples, 36 were obtained for physical examination purposes, and 68 had triggered the sample review rules^24^. This study was approved by the Ethics Committee of Ruijin Hospital, with approval number 2021 (72). Informed consent was waived because the research utilized only de-identified residual samples obtained during routine clinical testing, and the study posed no additional risk to patients.

The 104 venous blood samples were collected in EDTA-K2 vacuum blood collection tubes, from which peripheral blood smears were prepared within 4 h of collection with an SC-120 automated slide makers (Mindray, China) and then analysed with an MC-100i digital cell morphology analysers (Mindray, China), which captured 200 leukocyte images per smear. The leukocyte images of the 104 samples captured by MC-100i were collected to form a cell image library, which was saved on a laptop computer (Lenovo, China).

### Establishment of the gold standard for cell classification

The gold standard for cell classification in this study was established in accordance with the guideline Reference Leukocyte (WBC) Differential Count (Proportional) and Evaluation of Instrumental Methods (H20-A2)^25^, and a ground truth dataset was constructed using a three-expert joint interpretation process.

All cells were independently classified by three senior morphological experts from The First Bethune Hospital of Jilin University—a top-tier Grade A tertiary hospital with 5900 beds and a national authority in hematological disease diagnosis and treatment—using a drag-and-drop cell image workflow. These experts held associate senior titles or above, had 15–35 years of clinical laboratory experience in peripheral blood cell morphology, and were members of The Chinese Society of Laboratory Medicine (CSLM).

The specific process was as follows: the three experts performed cell image classification on 104 samples. Discrepancies were resolved by 2:1 majority consensus; samples with a 1:1:1 disagreement were excluded after verifying root causes as image acquisition or shooting technical defects. This ensured no morphologically ambiguous cells remained, and a ground truth dataset containing 19,174 qualified cells was successfully established.

To ensure gold standard reliability, multiple optimization measures were implemented: ① Experts were recruited from an authoritative hematology hospital to guarantee interpretation professionalism; ② Unified training on guidelines and research standards was conducted pre-interpretation to standardize morphological thresholds and reduce subjective differences; ③ The Kappa coefficient was used to quantify consistency, with a value of 0.98 among the three experts, indicating excellent agreement; ④ Borderline cases were adjudicated by integrating patients’ clinical information (e.g., hematological diagnosis results) to avoid systematic biases.

### AI pre-classification and morphologist evaluation process

Subsequently, the MC-100i performed pre-classification on all cells in the cell image library using a Convolutional Neural Network (CNN), with the outcomes documented as artificial intelligence classification results. After removing the identification labels provided by the AI pre-classification system, each laboratory morphologist independently classified the cells in the cell image library using the aforementioned drag-and-drop interface on the same laptop computer. Subsequently, the MC-100i performed pre-classification on all cells in the cell image library using a CNN, with the outcomes documented as artificial intelligence classification results. Ultimately, 15 laboratory morphological examination physicians from five Tertiary Grade A Hospitals in Shanghai, Guangzhou, and Wuhan were selected, including 4 with junior professional titles, 8 with intermediate professional titles, 1 with an associate senior title, and 2 with senior titles. Prior to the formal experiment, all participating physicians received unified pre-training, covering the experimental workflow, cell classification criteria (in accordance with International Council for Standardization in Hematology (ICSH) recommendations and H20 guideline^25^, the operation of the drag-and-drop classification interface. After removing the identification labels provided by the AI pre-classification system, each laboratory morphologist independently classified the cells in the cell image library using the aforementioned drag-and-drop interface on the same laptop computer. Strict controlled conditions were implemented during the process: referring to clinical routine morphological examination specifications and the H26 guideline^26^, the independent interpretation time for each sample was strictly controlled within 15–20 minutes (each sample requires classification and counting of 200 leukocytes). After completing independent interpretation of 5 consecutive samples, each expert was required to take a mandatory rest interval of 10–15 minutes. In addition, each expert was prohibited from continuously completing more than 15 samples per day, and special personnel supervised the entire process to ensure all conditions were strictly followed.

**Fig. 5:**
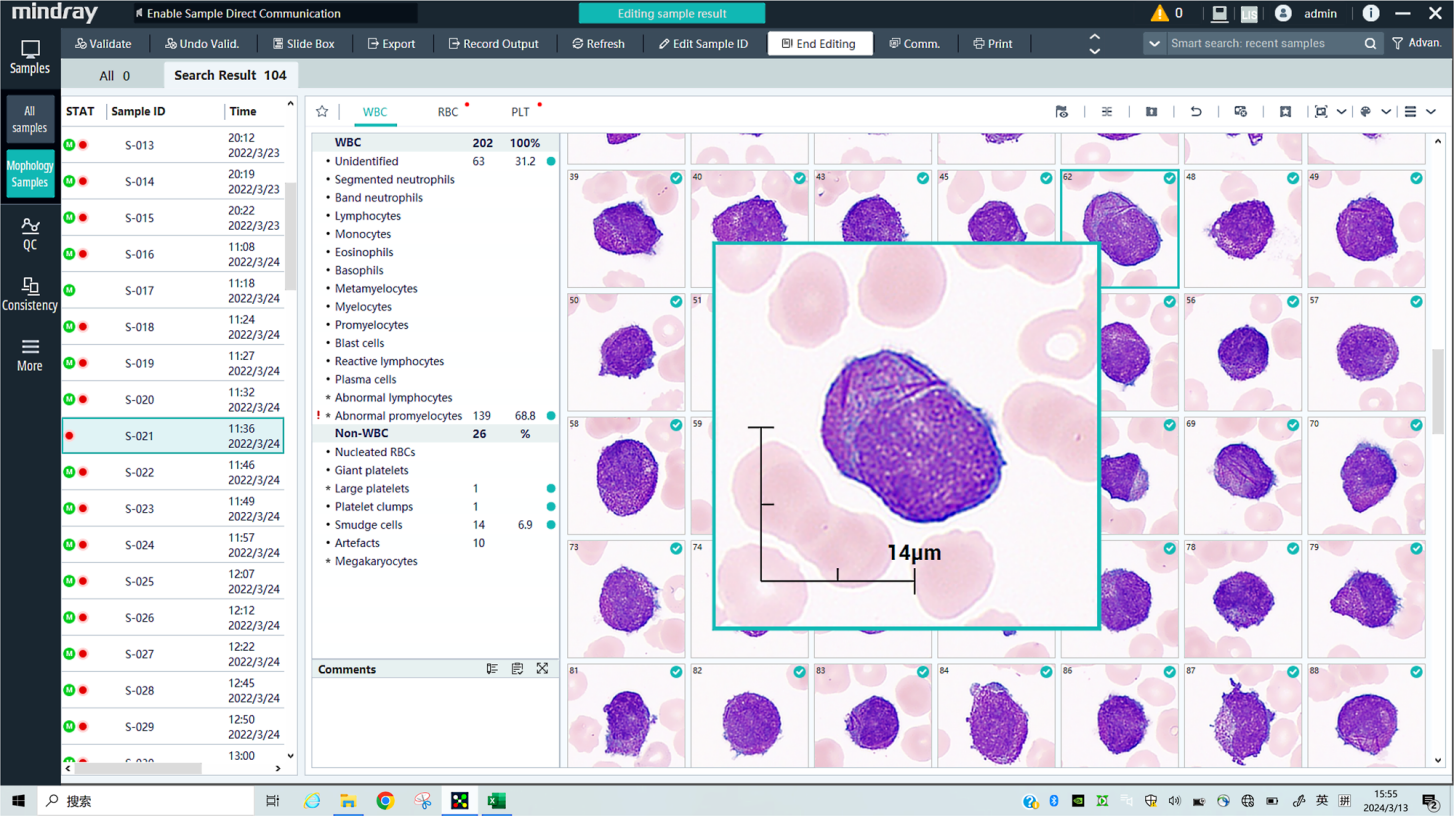
The display interface of MC-100i’s cell classification system on a laptop computer. The interface integrates a left panel displaying patient sample results and AI-generated leukocyte classification results, a central grid of sample cell images designed for drag-and-drop manual sorting, and an enlarged 14 μm-scaled view of a single cell (activated by clicking a cell).

**Fig. 6:**
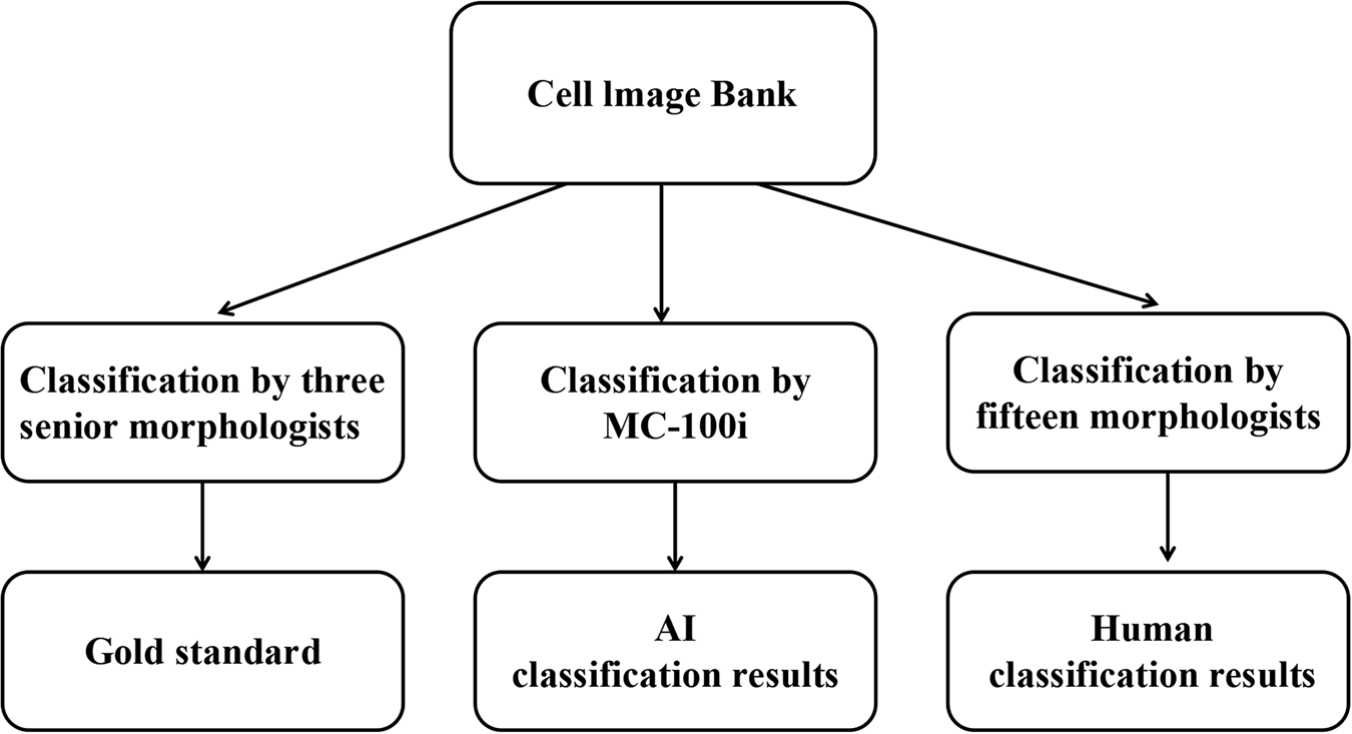
The workflow of cell classification. This flowchart outlines the study’s experimental pipeline, starting from a cell image bank that feeds into three parallel classification pathways: manual review by three senior morphologists to generate standard reference answers, automated analysis by the MC-100i AI system to produce AI classification results, and manual assessment by fifteen morphologists to obtain human classification results, enabling direct performance comparison between the AI system and human.

The morphologists were instructed to identify leukocytes from the image taken for each blood smear according to International Council for Standardization in Hematology (ICSH) recommendations^25,27^, classify them into one of 14 categories of leukocytes or nucleated erythrocytes, and record the results (the classification interface is shown in Fig. 5, and the detailed process is illustrated in Fig. 6). Normal cells included band neutrophils (*n* = 622), segmented neutrophils (*n* = 7824), eosinophils (*n* = 291), basophils (*n* = 223), lymphocytes (*n* = 5887) and monocytes (*n* = 1173). Blast granulocytes, promonocytes, and prolymphocytes were grouped as blast cells, immature eosinophils and mature eosinophils were grouped as eosinophils, and immature basophils and mature basophils were grouped as basophils. Abnormal cells included blasts (*n* = 1345), promyelocytes (*n* = 75), myelocytes (*n* = 492), metamyelocytes (*n* = 226), reactive lymphocytes (*n* = 54), abnormal lymphocytes (*n* = 101), abnormal promyelocytes (*n* = 444), plasma cells (*n* = 85), and nucleated erythrocytes (*n* = 332).

### Statistical analysis

Data analysis was conducted using Analyse-it (v5.11) and Excel 2013 software^16^. The image analysis was performed exclusively using Python(v3.7). For each cell type, the accuracy of the cell pre-classification, in percentage (%), was calculated as the number of correctly pre-classified cells of that type/total number of pre-classified cells of that type × 100%. Similarly, the sensitivity (%) was calculated as the number of correctly pre-classified cells of that type/total number of that type of cell after manual review × 100%, and the specificity (%) was calculated as the total number of cells other than that type that were correctly classified after manual review/total number of cells other than that type after manual review × 100%. On this basis, additional analyses, including rate difference confidence interval analysis, F1 score analysis, SHAP analysis^28^ and Grad-CAM analysis^29^ were carried out. Among them, for the rate difference confidence interval analysis (aimed at quantifying and verifying the difference in classification performance between AI and each morphologist), a superiority margin of 0 was preset. The specific judgment criteria were as follows: the rate difference was calculated as (AI accuracy - morphologist accuracy), and if the lower limit of the 95% confidence interval of the rate difference was higher than the superiority margin of 0, the difference in classification results between AI and the morphologist was considered statistically significant.

## Supplementary information

Supplementary Information V3

## Data Availability

The datasets generated and analysed during the current study are not publicly available due to privacy and ethical restrictions imposed by the Ethics Committee of Ruijin Hospital, as the datasets contain sensitive clinical information, but are available from the corresponding author on reasonable request.

## Code availability

This study evaluated the commercially available AI image-reading function integrated in the MC-100i hematology analyzer. No custom computer code or scripts were developed or used by the authors to generate the results reported in this manuscript. The underlying AI algorithm is proprietary to the manufacturer of the MC-100i analyzer. Requests for access to relevant algorithm details may be directed to the corresponding author for coordination with the manufacturer.
