# Supplementary material for: Evaluating AI in leukocyte classification: performance of the AI system against 15 morphology experts
Source: NPJ Digit Med. 2026 Apr 11;9:460. doi: 10.1038/s41746-026-02601-w (PMC13269485; doi:10.1038/s41746-026-02601-w)
Supplement: Supplementary file 1 — Supplementary Information V3 [file 41746_2026_2601_MOESM1_ESM.docx]

Supplementary Table S1. - F1 Score Table of Cell Classification by Different Interpretation Personnel.

| Personnel Type | Segmented neutrophils | Band neutrophils | Lymphocytes | Monocytes | Eosinophils | Basophils | Metamyelocytes | Myelocyteses | Promyelocytes | Blast cells | Reactive lymphocytes | Plasma cells | Abnormal lymphocytes | Abnormal promyelocytes | Nucleated RBCs |
| --- | --- | --- | --- | --- | --- | --- | --- | --- | --- | --- | --- | --- | --- | --- | --- |
| Human_15 | 0.999 | 0.991 | 0.983 | 0.959 | 0.974 | 0.973 | 0.910 | 0.902 | 0.529 | 0.942 | 0.697 | 0.976 | 0.130 | 0.882 | 0.996 |
| AI | 0.957 | 0.951 | 0.981 | 0.921 | 0.991 | 0.958 | 0.653 | 0.798 | 0.569 | 0.924 | 0.694 | 0.803 | 0.620 | 0.866 | 0.979 |
| Human_14 | 0.942 | 0.957 | 0.976 | 0.939 | 0.976 | 0.943 | 0.565 | 0.813 | 0.406 | 0.863 | 0.656 | 0.911 | 0.026 | 0.836 | 0.982 |
| Human_13 | 0.935 | 0.962 | 0.973 | 0.926 | 0.976 | 0.964 | 0.592 | 0.757 | 0.300 | 0.884 | 0.456 | 0.513 | 0.094 | 0.838 | 0.983 |
| Human_12 | 0.948 | 0.952 | 0.971 | 0.908 | 0.973 | 0.930 | 0.584 | 0.810 | 0.424 | 0.871 | 0.562 | 0.940 | 0.088 | 0.835 | 0.978 |
| Human_11 | 0.967 | 0.976 | 0.977 | 0.940 | 0.965 | 0.939 | 0.566 | 0.693 | 0.281 | 0.888 | 0.286 | 0.907 | 0.473 | 0.783 | 0.987 |
| Human_10 | 0.960 | 0.959 | 0.962 | 0.884 | 0.961 | 0.927 | 0.561 | 0.699 | 0.354 | 0.823 | 0.546 | 0.861 | 0.037 | 0.788 | 0.960 |
| Human_9 | 0.883 | 0.972 | 0.971 | 0.900 | 0.968 | 0.935 | 0.443 | 0.662 | 0.426 | 0.870 | 0.500 | 0.851 | 0.019 | 0.648 | 0.987 |
| Human_8 | 0.953 | 0.980 | 0.971 | 0.889 | 0.976 | 0.924 | 0.425 | 0.512 | 0.324 | 0.875 | 0.219 | 0.853 | 0.717 | 0.658 | 0.973 |
| Human_7 | 0.967 | 0.973 | 0.971 | 0.889 | 0.976 | 0.924 | 0.426 | 0.512 | 0.302 | 0.874 | 0.219 | 0.853 | 0.717 | 0.630 | 0.973 |
| Human_6 | 0.880 | 0.927 | 0.969 | 0.925 | 0.969 | 0.949 | 0.444 | 0.682 | 0.310 | 0.873 | 0.413 | 0.440 | / | 0.502 | 0.978 |
| Human_5 | 0.803 | 0.948 | 0.966 | 0.886 | 0.986 | 0.949 | 0.565 | 0.568 | 0.274 | 0.798 | 0.550 | 0.754 | 0.018 | 0.621 | 0.986 |
| Human_4 | 0.933 | 0.886 | 0.959 | 0.896 | 0.981 | 0.944 | 0.427 | 0.637 | 0.256 | 0.884 | 0.532 | 0.775 | 0.362 | 0.576 | 0.974 |
| Human_3 | 0.910 | 0.968 | 0.956 | 0.826 | 0.963 | 0.917 | 0.358 | 0.773 | 0.207 | 0.819 | / | 0.455 | / | 0.238 | 0.968 |
| Human_2 | 0.907 | 0.937 | 0.968 | 0.912 | 0.959 | 0.959 | 0.550 | 0.798 | 0.184 | 0.672 | 0.240 | 0.455 | 0.197 | 0.746 | 0.983 |
| Human_1 | 0.806 | 0.960 | 0.904 | 0.571 | 0.948 | 0.904 | 0.356 | 0.357 | 0.302 | 0.367 | 0.102 | 0.711 | 0.422 | 0.497 | 0.952 |

Supplementary Table S2. The number of cells in each type and the distribution of samples.

| No. | Cell types | Cell number | Cell distribution |
| --- | --- | --- | --- |
| 1 | Segmented neutrophils | 7824 | In 99 samples |
| 2 | Band neutrophils | 622 | In 77 samples |
| 3 | Lymphocytes | 5887 | In 103 samples |
| 4 | Monocytes | 1173 | In 92 samples |
| 5 | Eosinophils | 291 | In 72 samples |
| 6 | Basophils | 223 | In 62 samples |
| 7 | Metamyelocytes | 226 | In 30 samples |
| 8 | Myelocyteses | 492 | In 35 samples |
| 9 | Promyelocytes | 75 | In 14 samples |
| 10 | Blast cells | 1345 | In 33 samples |
| 11 | Reactive lymphocytes | 54 | In 26 samples |
| 12 | Plasma cells | 85 | In 4 samples |
| 13 | Abnormal lymphocytes | 101 | In 2 samples |
| 14 | Abnormal promyelocytes | 444 | In 7 samples |
| 15 | Nucleated RBCs | 332 | In 32 samples |
|  | Total Cells | 19174 | / |
